# Mesenchymal Stromal Cell Secretome for Post-COVID-19 Pulmonary Fibrosis: A New Therapy to Treat the Long-Term Lung Sequelae?

**DOI:** 10.3390/cells10051203

**Published:** 2021-05-14

**Authors:** Elia Bari, Ilaria Ferrarotti, Laura Saracino, Sara Perteghella, Maria Luisa Torre, Luca Richeldi, Angelo Guido Corsico

**Affiliations:** 1 Department of Drug Sciences, University of Pavia, Viale Taramelli 12, 27100 Pavia, Italy; elia.bari@unipv.it (E.B.); sara.perteghella@unipv.it (S.P.); 2 Center for Diagnosis of Inherited Alpha1-Antitrypsin Deficiency, Department of Internal Medicine and Therapeutics, Pneumology Unit IRCCS San Matteo Hospital Foundation, University of Pavia, 27100 Pavia, Italy; ilaria.ferrarotti@unipv.it (I.F.); corsico@unipv.it (A.G.C.); 3 Pneumology Unit IRCCS San Matteo Hospital Foundation, 27100 Pavia, Italy; l.saracino@smatteo.pv.it; 4 PharmaExceed S.r.l., 27100 Pavia, Italy; 5 Complex Operative Unit of Pneumology, Fondazione Policlinico Universitario A. Gemelli IRCCS, Catholic University of the Sacred Heart, 00168 Rome, Italy; luca.richeldi@policlinicogemelli.it

**Keywords:** SARS-CoV-2, COVID-19, pulmonary fibrosis, mesenchymal stem cells, extracellular vesicles, microvesicles, exosomes, secretome

## Abstract

To date, more than 100 million people worldwide have recovered from COVID-19. Unfortunately, although the virus is eradicated in such patients, fibrotic irreversible interstitial lung disease (pulmonary fibrosis, PF) is clinically evident. Given the vast numbers of individuals affected, it is urgent to design a strategy to prevent a second wave of late mortality associated with COVID-19 PF as a long-term consequence of such a devastating pandemic. Available antifibrotic therapies, namely nintedanib and pirfenidone, might have a role in attenuating profibrotic pathways in SARS-CoV-2 infection but are not economically sustainable by national health systems and have critical adverse effects. It is our opinion that the mesenchymal stem cell secretome could offer a new therapeutic approach in treating COVID-19 fibrotic lungs through its anti-inflammatory and antifibrotic factors.

The novel coronavirus, severe acute respiratory syndrome coronavirus 2 (SARS-CoV-2), has caused more than 117 million global cases and 2.6 million global deaths in the first year of the pandemic [1], with enormous economic and social derangements. In this unprecedented pandemic, serious efforts are being made worldwide. Regarding the preventive approach, good hygiene, social distancing and quarantine practices are applied to reduce the virus’s transmission. Advanced molecular biology and biotechnological approaches have been exploited to develop and launch effective vaccines against SARS-CoV-2 [2]. About the curative approach, an array of drugs are being studied for COVID-19 treatment in hundreds of clinical trials worldwide, as recently reviewed [3]. Therefore, although the COVID-19 pandemic still represents a challenge, instruments are available to prevent and treat the acute phase’s infection. However, very little has been done to solve the long-term consequence of such a devastating pandemic.

To date, more than 100 million people worldwide have recovered from COVID-19. Unfortunately, although the virus is eradicated in such patients, fibrotic interstitial lung disease (pulmonary fibrosis, PF) may become clinically evident in a large percentage of patients.

Generally speaking, PF can develop either following chronic inflammation or as a primary, genetically influenced and age-related fibroproliferative process, as in idiopathic pulmonary fibrosis (IPF). Previous coronavirus outbreaks have been associated with substantial post-viral fibrosis and physiological impairment; therefore, given the pandemic’s scale for SARS-CoV-2 infection, the global burden of fibrotic lung disease will probably increase considerably in the next years [4]. Indeed, PF is a recognized sequela of acute respiratory distress syndrome (ARDS), and available data indicate that about 40% of patients with COVID-19 develop ARDS, with 20% of ARDS cases being severe [5]. A substantial proportion of patients who develop ARDS will experience residual long-term impairment of lung function and computed tomography (CT) evidence of pulmonary fibrosis, with anterior reticulation the dominant abnormality in as many as 85% of survivors [6,7,8]. The extent of reticulation on CT correlates with the quality of life and lung function measures of pulmonary restriction, such as forced vital capacity (FVC) and the diffusion of the lung for carbon monoxide (DLCO), with approximately 25% of survivors exhibiting physiological evidence of restrictive lung disease [9]. Moreover, mediastinal emphysema, multiple bullae, giant bulla and pneumothorax developed during COVID-19 pneumonia are frequent, even in patients not treated with mechanical ventilation [10]. Finally, the average age of patients hospitalized with severe COVID-19 appears to be older than that seen with Middle East Respiratory Syndrome (MERS) or Severe Acute Respiratory Syndrome (SARS). In inflammatory lung disorders, such as those associated with autoimmune disease, advancing age is a risk factor for PF development. Even though long-term data are still lacking, these suppositions seem confirmed: Pan and colleagues observed fibrous stripes in 17% of patients in the early phase of the disease [11], while Ye and colleagues observed PF evidence in 23.7% of patients [12]. According to other reports, PF is estimated to affect around one-third of the patients hospitalized with SARS-COV-2 [13,14]. Given the vast numbers of individuals affected by COVID-19, even rare complications will have major public health effects, and especially the burden of PF after COVID-19 recovery could be substantial [15,16]. Nevertheless, it is important to note that, contrary to irreversible IL, in post-severe acute respiratory syndrome, CT findings considered to denote fibrosis at initial CT follow-up continue to regress in the longer term [17]. It is therefore urgent to design a strategy to prevent a second wave of late mortality associated with COVID-19 PF as a medium/long-term consequence of SARS-CoV-2.

Available antifibrotic therapies, namely nintedanib and pirfenidone, have broad antifibrotic activity regardless of etiology, and these drugs might have a role in attenuating profibrotic pathways in SARS-CoV-2 infection. Before 2019, nintedanib and pirfenidone had been studied exclusively in IPF. They appear to inhibit fibrogenesis across a wide range of pulmonary disorders, including pathogenetic profibrotic pathways driven by immune dysregulation, which might have similarities to those pathways in SARS-CoV-2 infection [18]. Currently, only one phase III clinical trial is recruiting patients to assess the effectiveness of nintedanib for COVID-19 PF (compared to placebo) on 250 patients (NCT04541680, http://www.clinicaltrials.gov (accessed on 9 March 2021)). However, therapy with nintedanib or pirfenidone is not economically sustainable by national health systems (NHS) for the many patients expected in the coming years (the mean adjusted annual costs for patients treated with pirfenidone and nintedanib are about $40,000 and $29,000, respectively [19]). Furthermore, the critical adverse effects (admittable for IPF due to the pathology’s progressive nature with a fatal outcome of five years after diagnosis) may not be acceptable in a non-progressive pathological context and with a less poor (presumed) prognosis than for IPF.

After many successful preclinical studies, treatment with mesenchymal stem cells (MSCs) is considered a potential new therapy for PF [20]. A recent clinical trial revealed that a high cumulative dose of MSCs is safe and well-tolerated by patients with IPF with a rapid lung function decline. Twenty patients with FVC ≥ 40% and DLCO ≥ 20% with a decline of both >10% over the previous 12 months were treated with MSCs (2 × 10^8^ cells) every three months vs. placebo. There were no significant adverse effects after the administration of MSCs in any patients. With MSC therapy, the 6-min walk distance significantly improved in 13 weeks, DLCO in 26 weeks and FVC in 39 weeks vs. placebo. FVC for 12 months with MSCs increased by 7.8% from baseline, whereas it declined by 5.9% with placebo [21]. The non-progressive and less poor (presumed) prognosis for COVID-19 PF suggests that MSC treatment will be even more effective than for IPF.

Although those findings might move future trials toward a new step in stem cells transplantation, MSC therapy is still linked to logistical problems (freezing and transport of cells from the production site to the hospital facilities) and to the need to have a laboratory available for cell manipulation where the infusion is performed. In addition, MSCs effectiveness may be hampered by low-engraftment or by a hard-to-control batch-to-batch variability [22]. In this regard, the use of the MSC secretome (the protein and extracellular vesicles secreted by MSCs) could be a valid substitute. The MSC secretome contains both soluble proteins (cytokine, chemokine and growth factors) and nano/microstructured extracellular vesicles (EVs), and it is reported to be active for fibrotic lung regeneration through anti-inflammatory and antifibrotic factors [23,24,25,26,27,28,29]. We recently proposed a freeze dried-secretome formulation (lyosecretome) for lung regeneration [30,31] and, as a new therapeutic approach in treating COVID-19 pneumonia [26], due to the broad pharmacological effects it shows, including anti-inflammatory, immunomodulatory, regenerative, pro-angiogenic and antifibrotic properties. For the same reasons, the MSC secretome could be a valid option in the treatment of post-COVID-19 PF. Indeed, it has been demonstrated that the effects of MSCs in PF are related to their ability to produce many biologically active substances with anti-inflammatory, immunosuppressive and angiogenic properties. In this regard, in vivo experiments revealed that the addition of the MSC secretome to bleomycin-injured rats improved lung architecture and reduced α-SMA and collagen content [27]. Similarly, it has been demonstrated that the inhalation of MSC EVs could attenuate and resolve bleomycin- and silica-induced PF by decreasing collagen accumulation and myofibroblast proliferation, reestablishing typical alveolar structure [28]. Unfortunately, few studies have compared the biological effects of secretome or EVs with parental MSCs, with contradictory results. According to Elliot and colleagues, MSC-derived exosomes appear to have similar therapeutic efficacy in a rat model of bleomycin-induced PF compared to whole-cell MSC infusions [32]; conversely, Choi and colleagues found the EV treatment was less effective than MSC treatment [24]. It is likely that the dosage of MSC secretome should be adjusted, considering that whole-cell MSC adaptation to the lung conditions and continuous release of bioactive factors are not easily replicable with one single MSC secretome administration [33]. In addition, the possibility to “educate” MSCs to produce a secretome with the best activity for PF treatment should be considered [31].

It is worth mentioning that the use of secretome is linked to advantages: (i) the availability of an on-the-shelf product with the same effectiveness as MSCs; (ii) ease of storage/transport/use in outpatient settings; and (iii) safer than replicating live cells (potentially tumorigenic). Specifically for safety, a recent review of the literature reveals that the administration of secretome or EVs by intravenous injection is safe, as no adverse effects have been reported [31]. Concerns regarding the safety after inhalation may arise due to the presence of cytokines and other many proteins in the MSC secretome, which may induce an immunogenic reaction, even if this side effect has not been revealed in animal models [28]. Phase II clinical studies are therefore needed to define the dosage, timing and frequency of administration. Finally, scalable and GMP-compliant isolation processes to make large doses of MSC secretome available to physicians exist [30,33,34,35], as well as the possibility to formulate it into injectable or inhalable dosage forms [31]. Compared with antifibrotic therapy (and even whole-cell MSC therapy), the costs of MSC secretome are expected to be lower, which is important in treating the consequence of a pandemic. Again, by defining dosage, timing and frequency of administration in phase III clinical trials, this aspect will be confirmed or not.

Despite no clinical trials currently investigating MSCs or their secretome in the treatment of COVID-19 PF, it is our opinion that they may represent a well-suited approach for treating patients with COVID-19 PF. Preliminary results and the scientific background encourage further investigation on MSC secretome treatment for COVID-19 PF with well-designed clinical trials under regulatory authorities’ control.
